# Microbial metabolic engineering: An annotated selection of World Wide Web sites relevant to the topics in microbial biotechnology

**DOI:** 10.1111/1751-7915.14070

**Published:** 2022-04-28

**Authors:** Lawrence P. Wackett

**Affiliations:** ^1^ Department of Biochemistry, Molecular Biology & Biophysics, BioTechnology Institute University of Minnesota St. Paul MN 55108 USA

## Metabolic engineering for flavonoids

### https://www.frontiersin.org/articles/10.3389/fbioe.2020.589069/full

Flavonoids are considered useful as nutraceuticals and pharmaceuticals. This article reviews engineering bacteria for the production of various flavonoid compounds.

## Metabolic engineering for nutraceuticals

### https://microbialcellfactories.biomedcentral.com/articles/10.1186/s12934‐019‐1096‐y

While nutraceuticals have traditionally been isolated from plants, this large global market is now increasingly being met using microbial platforms for production.

## Metabolic engineering for aromatic compounds

### https://microbialcellfactories.biomedcentral.com/articles/10.1186/s12934‐019‐1090‐4

This review highlights microbial engineering for the production of aromatic chemicals, largely derived from shikimate and aromatic amino acids.

## Physiological limitations in microbial metabolic engineering

### https://www.nature.com/articles/s41579‐021‐00600‐0

This review discusses aspects of microbial metabolic networks and regulations that might impose obstacles to metabolic engineering.

## Metabolic engineering for C1 feedstocks

### https://www.nature.com/articles/s41589‐021‐00836‐0

Due to the relatively low cost of natural gas as a carbon source, there is significant interest to engineer synthetic C1‐utilizing microorganisms to use as platforms for making industrial chemicals.

## Metabolic engineering for lycopene

### https://pubs.acs.org/doi/10.1021/acs.jafc.0c06020

Lycopene is valuable as a natural colorant and health supplement. This study looked at the production of lycopene in different microbial hosts.

## Metabolic engineering protocols

### https://link.springer.com/book/10.1007/978‐1‐4939‐9142‐6

This compendium contains multiple entries of protocols relevant to microbial metabolic engineering, production and analyses.

## Metabolic engineering strategies

### https://pubs.rsc.org/en/content/articlehtml/2020/cs/d0cs00155d

This is a broad review article on tools and strategies for metabolic engineering of microbes for chemical production.

## Bacteria chassis for metabolic engineering

### https://sfamjournals.onlinelibrary.wiley.com/doi/10.1111/1751‐7915.13292

This review article discusses traditional metabolic engineering host bacteria such as *Escherichia coli, Bacillus* spp and *Pseudomonas* spp. It also considers alternative host genera such as *Roseobacter* and *Halomonas*.

## Rewiring metabolic network

### https://academic.oup.com/jimb/article/48/9‐10/kuab040/6313286

Using lignocellulose as a feedstock will be more efficient with the ability to co‐utilize mixed carbon sources. This article discusses that strategy.

## Metabolic engineering for higher alcohols

### https://journals.asm.org/doi/10.1128/mBio.01524‐14

Alcohols with more carbon atoms than ethanol are not produced in high yield naturally. Metabolic engineering is being examined to produce higher yields.

## Metabolic engineering for L‐alanine

### https://academic.oup.com/jimb/article/49/2/kuab057/6354787

This article discusses an anaerobic fermentation for the production of L‐alanine, an amino acid important in foods and medicines.

